# Istore: a project on innovative statistical methodologies to improve rare diseases clinical trials in limited populations

**DOI:** 10.1186/s13023-024-03103-2

**Published:** 2024-03-02

**Authors:** Stefanie Schoenen, Johan Verbeeck, Lukas Koletzko, Isabella Brambilla, Mathieu Kuchenbuch, Maya Dirani, Georg Zimmermann, Holger Dette, Ralf-Dieter Hilgers, Geert Molenberghs, Rima Nabbout

**Affiliations:** 1 Institute of Medical Statistics, RWTH Aachen University, Pauwelsstrasse 19, 52074 Aachen, Germany; 2 Institute of Statistics, Ruhr-University Bochum, Universitätsstraße 150, 44801 Bochum, Germany; 3 I-BioStat, Data Science Institute, Hasselt University, Martelarenlaan 42, 3500 Hasselt, Belgium; 4 I-BioStat, KU Leuven, Kapucijnenvoer 35, 3000 Leuven, Belgium; 5 Institut des Maladies Gènètiques Imagine-Necker Enfants malades Hospital, 24 Boulevard du Montparnasse, 75015 Paris, France; 6 Necker Enfants malades Hospital, 149 Rue de Sèvre, 75015 Paris, France; 7 Dravet Italia Onlus – European Patient Advocacy Group (ePAG) EpiCARE, 37100 Verona, Italy; 8 Department of Surgical Sciences, Dentistry, Gynecology and Pediatrics, Research Center for Pediatric Epilepsies, University of Verona, Via S. Francesco, 22, 37129 Verona, Italy; 9 Team Biostatistics and Big Medical Data, IDA Lab Salzburg, Paracelsus Medical University, Strubergasse 21, 5020 Salzburg, Austria

**Keywords:** Bias assessment with multiple endpoints, Finite populations, Multiple endpoints, Natural history modelling, Rare disease clinical trials, Similarity of subgroups

## Abstract

**Background:**

The conduct of rare disease clinical trials is still hampered by methodological problems. The number of patients suffering from a rare condition is variable, but may be very small and unfortunately statistical problems for small and finite populations have received less consideration. This paper describes the outline of the iSTORE project, its ambitions, and its methodological approaches.

**Methods:**

In very small populations, methodological challenges exacerbate. iSTORE’s ambition is to develop a comprehensive perspective on natural history course modelling through multiple endpoint methodologies, subgroup similarity identification, and improving level of evidence.

**Results:**

The methodological approaches cover methods for sound scientific modeling of natural history course data, showing similarity between subgroups, defining, and analyzing multiple endpoints and quantifying the level of evidence in multiple endpoint trials that are often hampered by bias.

**Conclusion:**

Through its expected results, iSTORE will contribute to the rare diseases research field by providing an approach to better inform about and thus being able to plan a clinical trial. The methodological derivations can be synchronized and transferability will be outlined.

## Background

Currently, around 30 million people in Europe suffer from one of the around 7000 distinct Rare Diseases (RD). These diseases differ in prevalence, though most of them are very rare. It is therefore necessary to adopt a finite population sampling framework, unlike in non-rare conditions, where it is acceptable to think of a clinical trial as being sampled from an infinitely large population.

The iSTORE project on innovative statistical methodologies to improve rare diseases clinical trials in limited populations starts from acknowledging that there are hurdles for implementing an efficient clinical trial to evaluate new treatments in RD. Key such hurdles encompass insufficient knowledge about the natural disease course, uncertainty has how to compose a suitable primary outcome variable, optimizing the design for sensitivity to treatment effect, for example by linking the selection of a primary outcome measure to bias mitigating tools, and uncertainty as to how to show similarity of treatment effects across subgroups. These problems are general in RDs, although to a different extent from disease to disease. Thus, formulating solutions in terms of adequate statistical tools provides important contributions to RD research and reflects the ambitions of the International Rare Diseases Research Consortium (IRDiRC) [1], as well as the rare disease moonshot initiative [2]. In iSTORE, a toolbox of highly transferable methods will be developed along the use case of the Dravet Syndrome. Dravet is a prototype disease of developmental and epileptic encephalopathies (DEE), addressing the aforementioned challenges. However, iSTORE’s developments are not limited to Dravet Syndrome but are aimed to be highly transferable to other diseases as well.

**Fig. 1:**
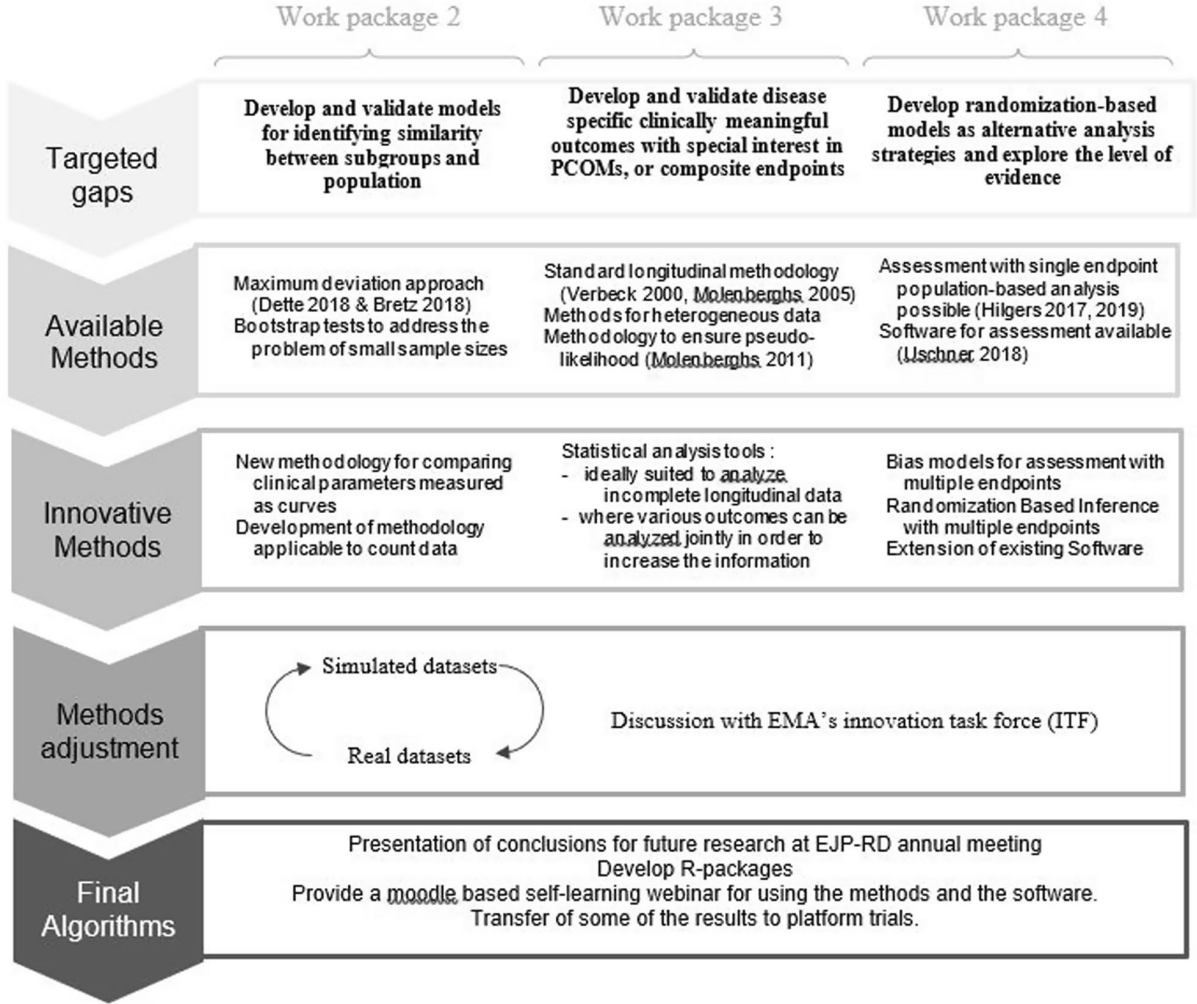
Objectives, organization, and workflow of the methodological developments in the iStore project: the project is organized in three methodological work packages. Each of them will provide innovative statistical methods suitable for RD

The iSTORE project is divided into four work packages. Work package 1 deals with the administrative and organizational work within the iSTORE project. Work packages 2, 3, and 4 focus on the development of innovative methodological approaches. Figure 1 provides an overview of the objectives, organization, and work flow of these methodological work packages. The paper is organized in six sections that describe the clinical problem, provide an overview of the project’s methodological approaches and the challenges following the workstream in Fig. 1.

In “Clinical problem” section, we describe the clinical problem and data source. The data will be used for modeling the natural history of seizures and identifying similarity between subgroup and populations in “Modelling natural history data of DS” and “Identifying similarity between subgroup and the population”. In “Statistical analysis of multiple endpoints” section, innovative methods for dealing with multiple endpoints are described. The last section is dedicated to evaluating the level of evidence from RCTs with multiple endpoints. In the discussion we focus on the expected results and impact.

## Clinical problem

*Challenges in rare diseases and specifically in developmental and epileptic encephalopathies (DEE) research:* DEE are rare diseases characterised by their low prevalence [3], clinical, and genetic heterogeneity [4], complexity, and multifaceted features. The patients suffer from refractory epilepsy along with several other neurodevelopmental, psychiatric, and motor comorbidities [5, 6]. Developmental and epileptic Encephalopathies (DEE) are an epitome of rare diseases elucidating various of these clinical trials hurdles. A general challenge in DEEs research include patient heterogeneity, as they do not share a unique homogeneous phenotype, nor genotype [4], making homogeneous subgroups too small and impending choosing uniform endpoints, ending with subgroups with different therapeutic profiles and different product development requirements [7]. Other challenges are encountered while conducting randomized clinical trials in rare diseases following the regulatory requirement of a high level of evidence. These obstacles are predominantly related to the small number of subjects, a hurdle well exemplified by Gallin [8]. In their study, no less than 10 years are required to recruit 39 patients. Similarly, Rees [9] confirmed in December 2014 that 30.2% of RD CTs conducted between January 2010 and December 2012 were discontinued, with the most frequently reported reason being insufficient patient accrual. Other obstacles include the large spectrum of the phenotypes in RDs (although due to a single genotype) [10] and to a big gap in our knowledge of the natural history of the disease and patients reported outcome measures.

Due to the peculiarity of these DEEs, another challenge that emerges is the need to compare different clinically relevant subgroups and/or a subgroup with the whole population, specifically when assessing efficacy, safety, and tolerability of a newly developed treatment or drug. Comparisons and analyses of subgroups is an integral part of the clinical trials, and guidelines have been published in this regard [11]. But, unfortunately, in rare and ultra-rare diseases, given that analysis of subgroups selection within clinical trial datasets might not be informative, there is still this huge need for innovative methodologies of subgroups comparison. Additionally in DEE, there is frequently a requirement to evaluate similarity of profiles (such as, for example, number of seizures). Moreover, in view of the DEE’s nature, vigilance is required on specific features of the longitudinal profiles, such as, for example, seizures’ variability over a period or in relation to aging and missing data.

Another challenge in DEE is the endpoints’ true representability of different disease aspects. Therapies in DEEs, like for other epilepsies, are based mainly on anti seizure medications and are usually assessed through randomized controlled trials. Typically, the decrease over 50% of the mean seizure frequency compared to baseline is defined as primary endpoint [12–15], which is highly representative of seizure decrease but might be less meaningful for other symptoms of the disease. Families, patients, and physicians agree that the impact of these DEEs go beyond seizures [16–18] and trials for treatment evaluation should take into account other endpoints as well [19]. Consequently, the Food and Drug Administration (FDA) in 2009 [20] and the European Medicines Agency (EMA) in 2016 [21], have encouraged the concept of Patient Reported Outcomes (PROs) as self-assessment of affected individuals. Gradually, the use of PROs in clinical trials has increased significantly since 2005 [22–24]. PROs can be used to determine affected individuals’ experience, particularly concerning improvement or aggravation of subjective symptoms, to stratify participants, to refine clinical trial design and to illustrate the risk-benefit balance allowing to choose the personalized best treatment [24]. These seem particularly necessary to effectively evaluate the impact of treatments in the field of rare epilepsy but also, more generally, in the field of rare diseases. And to further support use of endpoints that target what really matters for affected individuals, regulatory agencies recently finished the guidance about the use of composite endpoints and PROs [25].

Nowadays, various perspectives of patient outcome assessment, including the clinical outcome assessment, patient reported outcome, the clinician reported outcome, the observer reported outcome, and performance rated outcome [26], are considered appropriate. Specifically in DEEs, a collection of these outcomes are important to map the manifold responses to a treatment. So, including multiple outcomes in a properly selected combination appears to be a promising solution but requires quantification of the impact of bias on the level of evidence, both on the overall composite endpoint as well as on the individual component endpoints. This potential solution is crucial, especially because proof of efficacy in DEE clinical trials for these pathologies is sometimes difficult to provide. Currently, only four among the more frequent rare epilepsies have been subject to orphan drug development, namely Dravet syndrome (estimated prevalence 1:100,000), Lennox-Gastaut syndrome (10:100,000), infantile spams syndrome (12:100,000), and Tuberous sclerosis complex (3:100,000) [27].

*Dravet syndrome (DS), a showcase of DEE:* Dravet syndrome is a prototype of DEE and is a perfect showcase for these DEEs as it embodies all the challenges encountered in this epilepsy syndromes group, and thus an ideal candidate to test and apply the different innovative statistical methodologies. The onset of DS is usually during the first year (range 2–20 months) in a previously healthy infant. The seizure types and characteristics vary with age. Initially, they are either hemiclonic febrile and afebrile seizures, often alternating sides from seizure to seizure, or focal to bilateral tonic-clonic and/or generalized clonic seizures, and they are often prolonged. In preschool years, other seizure types often appear (myoclonic, focal impaired awareness, atypical absences, atonic, tonic or tonic-clonic) and by adulthood, brief tonic-clonic seizures, often occurring during sleep are most characteristic [6]. The seizures are commonly triggered by low grade fever, illness, vaccination, fatigue, photic stimulation, and visual patterns, and they are characteristically worsened with sodium channel blockers. Besides different age dependent seizures, patients with Dravet Syndrome will suffer from major non-seizure manifestations that are also age dependent and that include neurodevelopmental manifestations (intellectual disability, language delay, etc.), psychiatric disorders (autism spectrum disorders, attention deficit hyperactivity disorder, etc.) sleep disturbance (insomnia and other sleep disorders) and motor symptoms (crouched gait and acquired orthopedic malformations) [28]. These symptoms affect patients to variable degrees, culminating into a heterogenous group with a wide spectrum of symptoms. Thus, subgroups can be identified with different age of seizure onset, different combination of symptoms and/or comorbidities, different severity level of the manifestations, possible different genetic basis (although the majority have SCN1A mutation $> 80\%$), or different genetic variant types in SCN1A. In each of these subgroups, with eventually a very small number of patients per group, different endpoints are of interest. Moreover, the similarities between subgroups as well with the whole population in terms of efficacy, safety, and tolerability of response to a new treatment needs careful reflection in the assessment. Additionally, it should be noted that Dravet Syndrome is a lifelong disease with evolution of specific comorbidities over time [16]. This requires the collection of longitudinal data on all symptoms.

A common problem with clinical routinely collected longitudinal data is missing data for various reasons. Of course, the problems arising from missing data can be expected to exacerbate with smaller sample as well as population sizes, both of which are strongly related to rare diseases. Missing data may be due to patients’ non-compliance with their visit schedule, lost to follow up, incompleteness of information provided by patients and parents (who often provide proxy information), or physician’s under-reporting or mis-recording of information previously recorded by the parents in their diary and other notes. Missing data constitute a main complication in relation to the operational domain of the registry (32%) [29]. Assessing response to any treatment in Dravet Syndrome should take into account the improvement, stability, or worsening of all the seizure and non-seizure manifestations evaluated by physicians and technology (devices), but also and more critically reported by patients themselves and their caregivers [30, 31]. This suggests the need to include various disease aspects in a tailored clinical trial endpoint, which may take the form of patient reported outcomes (PRO).

### Data source - RESIDRAS register

We now briefly describe the data source, the RESIDRAS registry, that provides patient data on the Dravet Syndrom. The Associazione Dravet Italia Onlus [32], was established in Verona in 2010. The specific aim was to facilitate scientific research in Dravet Syndrome. For this purpose, a scientific committee created the national register “Registro Nazionale della Sindrome di Dravet e altre Sindromi correlate a mutazione dei gene SCN1A e PCDH19” (RESIDRAS). The Registry is an essential instrument to improve knowledge of the disease through the collection and systematic registration of patient information, with a constant flow of clinical data on patients. For every patient, there is at least a follow up of one year included. The RESIDRAS structure is used for the FP7 project “Research to improve diagnosis, prevention, and treatment in children with difficult to treat Epilepsy” [33]. The aim of the Registry is to acquire epidemiological, clinical, and genetic information and make this available to the scientific community, to national health services, and to patients and their families in order to support an adequate programme in the diagnostic-therapeutic-assistance areas. In fact, the collection of patient data affected by the mutation of the SCN1A and PCDH19 gene could help to evaluate the real dimension of the problem and promote research, with the ultimate objective of offering improved assistance.

The Italian Registry model has been developed by a working group consisting of expert clinicians, members of the Scientific Medical Committee, representatives of patient associations, experts in DS and registries and information technologies useful for their implementation. The working group, after having identified the main aims of the registry, developed its structure and established 11 headings: Anagraphic Data; Genetic Investigations; Family History; Personal History; Onset of Epileptic seizures, Seizures Follow-up; Neurological and Cognitive Follow-up; Therapy; Adverse events; Gait Analysis and Grow and Cardio Parameters sheet. Each of these headings is composed of a number of variables, mandatory and optional. Due to the positive experience, Dravet Italia Onlus set up an international registry called “Platform-RESIDRAS”. These two registries [34] have the same data set structure, but separate Coordinating Committees. The Registries have adopted the principles of Fairification (FAIR: Findable Accessible Interoperable Reusable). They are in line with the “Set of common data elements for Rare Diseases Registration”. This is the first practical tool released by the EU RD Platform that aims to increase the interoperability of RD registry data, given that they contain 14 out of 16 data elements common to all rare disease registries in Europe, a key asset for further research [35]. The Registries will use the following ontology codes: Unified Medical Language, Human Phenotype Ontology, Orphanet Rare Disease Ontology, HPO ORDO Ontological Module. The Registries have received a monitoring report in order to assess the workflow and GDPR compliance. It is included in the ENCEPP Databases - The European Network of Centres for Pharmacoepidemiology and Pharmacovigilance, a network coordinated by the European Medicines Agency. To date, a total of more than 650 patients have been entered in the registries, 400 in RESIDRAS and the other cases in the RESIDRAS platform. For the research within the iStore project we use an extract from the RESIDRAS registries at a fixed time point to develop and test the innovative methodological approaches. This data extract contains all patient data that has been collected in both registries up to this time point.

## Modelling natural history data of DS

In this section, the methodological approaches are described alongside the challenges to tailor these to modeling natural history data in Dravet Syndrome. To flexibly and adequately describe longitudinal outcomes, specifically when they consist of various components, potentially of differing data types, the standard linear and generalized linear mixed models [36, 37] may not be sufficient. One then should consider existing extensions that accommodate overdispersion as well as correlation with sufficient flexibility [38–40]. The joint analysis of several longitudinal sequences of different types was examined by Ivanova, Molenberghs, and Verbeke [41]. In addition, the possibility of excess zeroes in count outcomes (e.g., number of seizures) should be accommodated if needed [42–44]. Some, but not all models yield directly marginally interpretable mean and/or correlation functions. If this is not the case, additional computations are needed [45–47]. Additionally, assessing model fit and the impact of potentially influential subjects is imperative [48–50].

For small datasets or datasets with long sequences of repeated measures, and/or datasets with a large number of different variables measured longitudinally, computational issues may arise, in the sense that the conventional likelihood and Bayesian estimation algorithms may fail to converge or may take an inordinate amount of time to do so. Pseudo-likelihood and related methodology have proven to be very useful in this respect [37, Ch. 9, 12, 21 and 24]. A so-called pairwise fitting approach for high-dimensional longitudinal data was developed by Fieuws and Verbeke [51] and Fieuws et al. [52]; see also Molenberghs and Verbeke [37, Ch. 25]. Further approaches consist of splitting the sample in sub-samples, analysing each of these separately, and appropriately combining the results [53]. These computational tools can be applied simultaneously as well, as was done by Ivanova, Molenberghs, and Verbeke [41]. Splitting samples becomes a bit more involved when cluster sizes are unequal, e.g., because longitudinal sequences are of unequal length. One then needs to carefully consider a weighting scheme to apply [54]. In some cases, and somewhat less well known, one can fall back on closed-form estimators, which of course do not suffer from convergence issues [55].

One reason why sequences may be of unequal length is the occurrence of incomplete data [56]. Whereas full likelihood methods are broadly valid when data are incomplete, i.e., they can be applied when missing data are missing at random (MAR), meaning that missingness, given covariates and observed outcomes, does not further depend on unobserved outcomes, this is no longer true when pseudo-likelihood or other semi-parametric methods are used [57], in which case weighting procedures have to be applied, or alternatively the analysis has to be pre-processed using multiple imputation [58]. The trade-off between both approaches was investigated by Beunckens, Sotto, and Molenberghs [59]. An important advantage of multiple imputation is its efficiency and the fact that it helps stabilize computations. To examine the stability of the results, it is useful to apply multiple imputation on the one hand, and an ignorable analysis (for likelihood and Bayesian methods) or an inverse probability weighting based analysis (for other approaches). It is also possible to consider more than one imputation mechanisms, to investigate the robustness of the conclusions to imputation assumptions made.

Given that MAR cannot be established unambiguously based on the observed data, and hence that missing not at random (MNAR) (meaning that missingness, even given covariates and observed outcomes, still depends on unobserved outcomes), sensitivity analysis is called for as the capstone of any analysis of incomplete data [60]. Fortunately, a number of sensitivity analysis tools have been integrated with multiple imputation and are available, so that a set of sensitivity analyses under MNAR can be integrated seamlessly and compared with primary analyses under MAR.

Another frequently encountered issue regarding longitudinally observed count outcomes (e.g., seizure counts) might be the presence of a few very large counts and, generally, the presence of (extremely) skewed distributions. As a remedy, one may consider using a rank-based non-parametric approach (e.g., Burchett et al. [61], Dobler et al. [62]). In particular, a promising line of action would be the extension of these longitudinal non-parametric methods to also allow for missing data (e.g., Rubarth et al. [63]). Moreover, a closer examination of similar non-parametric approaches (e.g., generalized pairwise comparisons) in the context of the analysis of (multivariate) outcomes with (heavily) skewed distributions would be worthwhile. The research in this workstream can be based on previous work that has been conducted in the EBStatMax demonstration project, and on the substantial extensions for censored data [64] and suggestions for missing data [65].

Thus, our approach will investigate statistical analysis tools ideally suited to analyze incomplete longitudinal data, where various outcomes can be analyzed jointly, in order to increase the information extracted from the data.

## Identifying similarity between subgroup and the population

A very particular challenge in Dravet syndrome is that disease progression is specific to age. To identify disease progression age specific parameters, the natural disease course of Dravet subjects in the RESIDRAS registry will be modeled with highly flexible models. iSTORE will develop tools for improving treatment evaluation starting with clinical outcome formulation, identification of subgroups, and improving the design and analysis of clinical trials. Extending the results of Dette et al. [66] we will develop bootstrap tests for validating the similarity of response profiles (for example, a parameter measured over time) between rare diseases subgroups and the entire population. From a theoretical point of view, we will show that our approach provides a statistically valid procedure and we will empirically verify, via simulations and data analysis, that it is particularly suited for small sample sizes. We also expect that the new procedures will be more powerful than tests based on common statistical principles such as the union-intersection principle [67, 68]. Consequently, our methods are particularly well suited for studying rare diseases such as the Dravet syndrome. The techniques are quite general and thus applicable to varying notions of similarity, which makes our approach useful for a broad range of applications. We illustrate the method in the context of drug development, where we develop tests for the similarity between dose-response curves of a subgroup and the overall cohort of patients in a clinical trial with continuous or discrete responses. As another application, we will consider testing the similarity of class proportions, where the classes could, for instance, represent disability scores. Testing for similarities could serve as an effective approach to merging the international RESIDRAS platform registry with the Italian national RESIDRAS registry. This is relevant as both registries may be influenced by geographical heterogeneities. Overall, showing similarity (of any kind) between the overall population of patients and a particular subgroup can lead to a better understanding of the disease under consideration.

Thus our approach will investigate new methodology for comparing clinical parameters measured as curves between subgroups. Extension of the methods to count data is the next step of our development.

## Statistical analysis of multiple endpoints

Multiple clinically relevant endpoints can be tested for treatment effect between two groups, using different statistical analysis methods. In general, we distinguish between two approaches. In the first, each outcome is analyzed by separate statistical tests and the results are subsequently combined. In the second, the outcomes are first combined and subsequently analyzed in a single test. In either of these, it is very common to evaluate the treatment effect in each component of the multiple endpoint. This is sometimes even required by regulatory guidance.

The first approach encompasses the strategies of multiple primary endpoints, co-primary endpoints, and hierarchical testing. Testing multiple primary endpoints requires appropriate measures, such as Bonferroni corrections for multiple testing, to control the nominal type I error probability [69]. In contrast, the co-primary endpoint relies on an “all or none” decision rule, meaning that the treatment effect should be shown in all components of the endpoint simultaneously. The advantage is that then no type I error correction is required [25, 70]. Similarly, hierarchical testing, where the multiple components are tested in a pre-specified sequence according to clinical relevance, until the first non-significant test result, does not require type I error corrections [25, 70]. Major disadvantages of these first approach strategies are that a single combined treatment effect measure is not available and that the correlation between the endpoints is rarely considered, although some advancement has been made in this direction [71, 72].

The second approach comprises the concept of composite endpoints and multi-component endpoints. While in composite endpoints dimensionality is reduced by considering the occurrence of any of the components in the endpoint (for example, the first occurrence), in a multi-component endpoint the components are combined within a subject to a single score or rating [25]. In both cases, the examination of a treatment effect regarding a specific component is challenging. Examples of the latter include, but are not limited to, clinical indices and joint modelling.

A special case that links the hierarchical idea with combining the outcomes first, is the generalized pairwise comparisons (GPC) methodology [73–76], which results in what can be called a prioritized endpoint. GPC is an extension of the pairwise comparison version of the Mann–Whitney [77] or Gehan-Wilcoxon [78, 79] tests to multiple outcomes. The most frequently used GPC test compares the outcomes prioritized by clinical severity, in all possible pairs of subjects, with one subject from each treatment arm. If in a pair a difference is established on an outcome, the subsequent outcomes are not further considered. This results in an analysis that gives more weight to more severe outcomes. This contrasts the commonly applied time-to-first event analysis of a composite endpoint, where the time of the event is weighted rather than the severity of the event.

Importantly, in GPC any number and any type of outcome can be combined. Moreover, the correlation between these outcomes is captured, without explicitly modelling it [76]. Although it has been applied mainly in large sample trials, the exact permutation test of a GPC endpoint, has good small sample properties [76, 80, 81].

Interestingly, several extensions of the prioritized GPC exist. The non-prioritized GPC evaluates each of the outcomes in all possible pairs [76, 82], following the idea of the non-parametric O’Brien test [83]. Additionally, extensions to longitudinal outcomes [84] and for N-of-1 trials [85] are available. Covariate adjustment in GPC is feasible through stratification [86], although in small samples the stratum size needs careful attention. Interestingly, both regulatory agencies FDA and EMA, have endorsed the GPC analysis of a prioritized endpoint as primary analysis for the approval of the drug tafamidis in the rare disease amyloid cardiomyopathy [87].

Another technique, which was recently introduced is the multidomain responder index [88], which sums the scores of responses, defined by a clinically meaningful change across all components.

Traditional techniques for comparing two groups on multiple endpoints and showing an overall positive treatment effect on all components of the multiple endpoint are the ordinary least squares (OLS) and generalized least squares (GLS) tests of O’Brien [83]. Applying both test statistics to the standardised components of the multiple endpoint results in a weighted sum of individual t-statistics of the endpoint components. Here, the OLS test uses equal weights whereas the GLS test uses unequal weights utilizing the estimation of the correlation matrix between the endpoint components. According to Logan and Tamhane [89], the OLS test is the more preferable one because it converges faster to a limiting distribution than the GLS test statistic. It should be noted that these test procedures, in contrast to multiple test procedures such as the Bonferroni procedure, take the correlation structure of the multiple endpoint into account.

Since the distributions of the OLS and GLS test statistics under the null hypothesis are only approximate, they can lead to an inflation of the type I error, especially in clinical trials with small sample sizes. Läuter [90] improved both test procedures and developed a sum statistic that takes the factorial structure between the components of the endpoints into account, which follows an exact t-distribution under the null hypothesis. Thereby, methods of elliptically contoured distributions were used [91].

Our investigation is focused on comparing statistical methodologies suited for the analysis of multiple outcomes, potentially with longitudinal profiles, on their small sample behavior and sensitivity to discriminate treatment effects on individual or joint endpoints.

## Evaluation of level of evidence from RCTs with multiple endpoints

The fact that populations in rare diseases are limited in size suggest that tailored approaches are needed when conducting clinical trials, in particular with multiple endpoints. As rare diseases show a large spectrum of different symptoms, the use of multiple endpoints is considered advantageous. However, it is unclear whether the inclusion of multiple endpoints will result in a gain of level of evidence and how to measure and quantify the impact of bias on the level of evidence in this setting. In particular in a randomized two-arm parallel group design with multiple endpoints regulators recognized that clinical trials “may be subject to unanticipated, undetected, systematic biases. These biases may operate despite the best intentions of sponsors and investigators, and may lead to flawed conclusions. In addition, some investigators may bring conscious biases to evaluations” [25]. The impact of (allocation) bias on the trial can be quantified by comparing the actual biased type I error rate with the nominal significance level. Adopting this approach, we aim to implement a model to quantify the allocation bias effect on the result of a randomized clinical trial with multiple endpoints based on the convergence strategy of Blackwell and Hodges [92] and Proschan’s biasing policy [93].

The selection of the randomization procedure to mitigate bias and thus to increase the level of evidence is unknown in RD trials with multiple endpoints. We will consider a randomized single-center clinical trial in a two-arm parallel group design with a single time point, without interim assessment, and adopting analyses for different types of continuous multiple endpoints. We focus on different types of multiple endpoints as multiple primary endpoints and multi-component endpoints. The aim is to extend the bias model and the results for a single endpoint [94] to multiple endpoints and follow the recommendation in [95] with respect to the test statistics for multiple endpoints using population based inference. Firstly, we will derive an analytical model for the analysis procedures: Bonferroni, GLS-, OLS-Test and Läuter Test. This will be followed by a simulation study for the assessment of the level of evidence for multiple endpoints with population-based modelling in single center trials. Since rare disease clinical trials are often multi-center and international, the simulations will be extended to multi-center clinical trials in the next step. Therefore, a center effect term needs to be added to the model.

As the amount of allocation bias will vary between the “quality” of the endpoint components, our approach will allow the assessment of the level of evidence regarding different bias effects of the endpoint components. Thereby, we aim to provide a recommendation for the balance between the number of components in the multiple endpoints and the impact on the level of evidence under bias due to population-based modeling. When using sum statistics, as in the OLS and GLS tests, the number of endpoints influences the impact of allocation bias on test decisions. This is due to each endpoint introducing an endpoint-specific bias effect term to the test statistic [96]. Additionally, the approach will provide recommendations on the choice of randomization procedure based on the level of evidence. Our model approach will be embedded in the R-package randomizeR [97] to enable future analogous evaluations in similar disease areas. Overall, the derivations will aim to provide recommendations for the design of clinical trials with multiple endpoints in the field of rare diseases that increase the validity of the clinical trial by raising the level of evidence. Note that our approach can also be viewed as a basic concept that is transferable to platform trials as well.

In a second step, we will investigate a methodological approach for evaluating the evidence level of clinical trials with multiple endpoints in finite populations obtained by randomization-based inferences. The randomization-based inferences are particularly linked to the randomization procedure. Randomization-based models are not yet developed or embedded in a population based model approach. With multiple endpoints this becomes even more challenging.

Our investigation is focused on the development and implementation of a multi-component biasing policy enabling us to quantify the impact of bias on the test decision in a clinical trial with multiple endpoints.

## Discussion

The following discussion will elaborate on the summary provided in Table 1. The developments in iSTORE can be viewed as a Comprehensive Toolbox necessary to understand the course of a disease, to identify important subgroups, to assess multi-dimensional outcomes including patient-centered outcome measures (PCOMs), and to optimize bias mitigating trial design in rare diseases. It should be pointed out, that in general the tools may be used separately, and depending on the disease, modification might be necessary. However, the consortium is convinced of the high level of impact of the tools or implemented roadmap.

**Table 1:** Comprehensive toolbox for understanding and improvement of developmental and epileptic encephalopathies

What is known	What is expected	Impact
Methods for testing similarity between distinct groups of patients	Methods for testing similarity between subgroups and overall population	Powerful methods for improved understanding of rare diseases and allowing extrapolation of information between groups
Linear and nonlinear mixed effect modeling	Tailored approaches for modelling count data in limited populations to model longitudinal data of Dravet patients	Understanding of flexible modelling of natural history course data
Several statistical methodologies are capable of evaluating multiple endpoints in a single analysis	Recommendations of statistical methodologies to analyzing multiple endpoints of potential different data type in small sample trials	More efficient analysis of randomized trials with multiple endpoints in small sample trials
Quantification of impact of bias on the level of evidence in two arm parallel group design with single endpoint	Extension of the bias model to multiple endpoints corresponding to the analysis	More efficient randomized trials with multiple endpoints: Optimize trial designs with respect to level of evidence in case of multiple endpoints

### Expected results

The methodologies developed by this consortium will fill some important gaps commonly identified in trials on rare diseases with limited populations. The Dravet Syndrome serves as a use case for the methodological development, but—as frequently encountered in statistical methodologies—the tools provide a high level of transferability on a case by case basis. First, the methodologies will contribute to ameliorating the evaluation of efficacy of novel treatment regimes, targeting more precisely what matters for patients, and taking into account comparative evaluations in patient sub-populations. Secondly, the novel approaches will address a problem common to many longitudinal studies, namely the occurrence of incomplete data. This will allow for a valid statistical analysis of numerous cohorts studies whose conclusions are affected by large amounts of missing data. Therefore, the project team will propose solutions to methodological challenges that have not been satisfactorily addressed so far. On the other hand, in addition to the considerable impact in the field of biostatistical research, these new methodologies will also lead to improved analyses of concrete datasets from studies on rare epilepsies (e.g., a further prospective trial based on the RESIDRAS registry). Thus, the data of the RESIDRAS registry are essential to define i.e. meaningful endpoints and subgroups of similar disease characteristics for a future prospective clinical trial Moreover, through the involvement of some consortium partners in the EpiCare network, we will foster the idea of designing more efficient and patient-friendly therapeutic trials for rare epilepsies in the near future. The effectiveness of these therapeutic trials will focus on what matters most to patients and optimize design and analysis, thereby increasing the level of evidence. To facilitate the application of the proposed methods, open access software will be provided, along with corresponding instructional videos.

### Transferability

The new statistical methodologies developed in this project, although adapted to rare epilepsy trial approaches, can be easily transferred to almost all rare diseases. For example, counts are frequently used as primary outcomes not only in epilepsy, but also in epidermolysis bullosa (i.e., the reduction of blister numbers compared to baseline, see [98]). Moreover, heterogeneity of patients is often present (for example, due to different underlying genotypes). Therefore, it is valuable to have methods at hand that account for this heterogeneity by, for example, allowing for comparisons of subgroups to the overall population with a specific RD. Indeed, such approaches will be developed in the proposed collaborative project. Furthermore, in RD research in general, outcomes are often assessed longitudinally, in order to increase power in genuinely small populations, and to obtain conclusions about the natural history of the disease. However, the burden for patients when participating in a study is usually substantial, especially in rare diseases. Therefore, the amount of missing data is expected to be considerable, and merely excluding subjects with missing data might seriously affect the statistical analyses, given that the sample sizes are already low. Therefore, the new methodologies developed in the corresponding work packages on longitudinal data analysis in presence of missing data would not only resolve these issues with respect to rare epilepsies but serves as a solution to a problem that stems from the very nature of RD data. Last but not least, developing multi-component endpoints that truly capture what really matters to patients and their families is highly needed by patient representatives in any rare disease.

Operationally, the transfer of the project findings to other RD areas will be facilitated by the fact that several partners involved in the present project are already participating in the activities of various European networks on rare diseases (ERN), in particular ERN skin for rare skin diseases (G Zimmermann and G Molenberghs), the ERN EpiCare for rare and complex epilepsies (R Nabbout, I Brambilla, G Zimmermann), and EJP-RD (R Nabbout, RD Hilgers, G Molenberghs, G Zimmermann) and ERICA, the European Rare Disease Research Coordination and Support Action consortium (RD Hilgers). Moreover, as already mentioned above, the highly interdisciplinary composition of the project team (international partners from academia, clinical research, industry, and patient networks) and existing links to EMA and FDA will foster transferability and visibility of the project outcomes beyond the scope of rare epilepsies. Providing open-access and open-source software implementations along with manuals and tutorial videos will further enhance the use by various stakeholder groups within the rare diseases community. Of course, the project outcomes will also be circulated in the scientific community by publishing papers on the most important findings and presenting the novel methodological development at international conferences. This might also increase awareness of the methodological challenges related to rare diseases among biostatisticians, thereby attracting more researchers to dedicate their workforce to improving statistical techniques for analyzing RD data.

## Data Availability

The data that support the findings in this paper are available on request. RESIDRAS registry is not public available. Please contact IB.

## Abbreviations

DEE: Disease of developmental and epileptic encephalopathies
EMA: European Medicines Agency
ERICA: European rare disease research coordination and support action
ERN: European reference network
FDA: Food and Drug Administration
GDPR: General data protection regulation
GLS: Generalized least squares
GPC: Generalized pairwise comparisons
IRDiRC: International rare diseases research consortium
iSTORE: Innovative statistical methodologies to improve rare diseases clinical trials in limited populations
MAR: Missing at random
MNAR: Missing not at random
OLS: Ordinary least squares
PROs: Patient reported outcomes
RCT: Randomized controlled trial
RD: Rare Diseases
RESIDRAS: Registro Nazionale della Sindrome di Dravet e altre Sindromi correlate a mutazione dei gene SCN1A e PCDH19
